# Cuticular Wax Accumulation Is Associated with Drought Tolerance in Wheat Near-Isogenic Lines

**DOI:** 10.3389/fpls.2016.01809

**Published:** 2016-11-30

**Authors:** Jun Guo, Wen Xu, Xiaocong Yu, Hao Shen, Haosheng Li, Dungong Cheng, Aifeng Liu, Jianjun Liu, Cheng Liu, Shijie Zhao, Jianmin Song

**Affiliations:** ^1^National Engineering Laboratory for Wheat and Maize and Key Laboratory of Wheat Biology and Genetic Improvement in North Yellow and Huai River Valley, Ministry of Agriculture, Crop Research Institute, Shandong Academy of Agricultural SciencesJinan, China; ^2^State Key Laboratory of Crop Biology, College of Life Sciences, Shandong Agricultural UniversityTaian, China

**Keywords:** wheat, leaf cuticular wax, physiological traits, drought tolerance, selection criteria

## Abstract

Previous studies have shown that wheat grain yield is seriously affected by drought stress, and leaf cuticular wax is reportedly associated with drought tolerance. However, most studies have focused on cuticular wax biosynthesis and model species. The effects of cuticular wax on wheat drought tolerance have rarely been studied. The aims of the current study were to study the effects of leaf cuticular wax on wheat grain yield under drought stress using the above-mentioned wheat NILs and to discuss the possible physiological mechanism of cuticular wax on high grain yield under drought stress. Compared to water-irrigated (WI) conditions, the cuticular wax content (CWC) in glaucous and non-glaucous NILs under drought-stress (DS) conditions both increased; mean increase values were 151.1 and 114.4%, respectively, which was corroborated by scanning electronic microscopy images of large wax particles loaded on the surfaces of flag leaves. The average yield of glaucous NILs was higher than that of non-glaucous NILs under DS conditions in 2014 and 2015; mean values were 7368.37 kg·ha^−1^ and 7103.51 kg·ha^−1^. This suggested that glaucous NILs were more drought-tolerant than non-glaucous NILs (*P* = 0.05), which was supported by the findings of drought tolerance indices TOL and SSI in both years, the relatively high water potential and relative water content, and the low ELWL. Furthermore, the photosynthesis rate (*P_n_*) of glaucous and non-glaucous wheat NILs under DS conditions decreased by 7.5 and 9.8%, respectively; however, glaucous NILs still had higher mean values of *P_n_* than those of non-glaucous NILs, which perhaps resulted in the higher yield of glaucous NILs. This could be explained by the fact that glaucous NILs had a smaller *F_v_/F_m_* reduction, a smaller *PI* reduction and a greater *ABS/RC* increase than non-glaucous NILs under DS conditions. This is the first report to show that wheat cuticular wax accumulation is associated with drought tolerance. Moreover, the leaf CWC can be an effective selection criterion in the development of drought-tolerant wheat cultivars.

## Introduction

Drought is a serious problem in semi-arid and arid areas worldwide (Mardeh et al., 2006). It can cause losses in wheat grain yield of between 10 and 100% Foulkes et al., 2007; Li et al., 2011; FAO, 2013. Furthermore, climate change is likely to increase drought risk in the Twenty-first century in many parts of the world (Arnell, 2008). Hence, it is important to develop wheat cultivars with high drought tolerance in order to improve food security.

Wheat leaves with bloom or glaucous characteristics are coated with cuticular wax (Johnson et al., 1983; Tsunewaki and Ebana, 1999). To date, six genes controlling wax biosynthesis have been reported and are located on the following wheat chromosomes: *W1* and *IW1* on 2BS, *W2* and *IW2* on 2DS, *W3* on 2BS, and *IW3* on 1BS (Tsunewaki and Ebana, 1999; Adamski et al., 2013; Wu et al., 2013; Wang et al., 2014a; Zhang et al., 2015), but little is known about the functions of these genes. Previous studies showed that leaf cuticular wax can protect the plants against abiotic and biotic stresses, such as drought, UV and the wheat grain aphid (Blum and Ebercon, 1981; Shepherd and Wynne Griffiths, 2006; Wójcicka, 2015). Firstly, cuticular wax accumulated under drought stress in plants, such as tobacco, alfalfa, rice and wheat (Butler, 1996; Zhang et al., 2005, 2013; Cameron et al., 2006; Islam et al., 2009; Adamski et al., 2013; Wang et al., 2014a), indicating that it is related to drought tolerance. Secondly, leaf water potential decreased under drought tolerance (Zhang et al., 2013), but the effect of cuticular wax on water potential is never been studied. Thirdly, cuticular wax, as a photoprotective layer, can protect plants against UV radiation, but rare studies have been carried out to study the effects of cuticular wax on plant photosynthesis (Shepherd and Wynne Griffiths, 2006). Lastly, the effects of wheat leaf cuticular wax on wheat yield under drought stress have never been studied using wheat near-isogenic lines (NILs) with and without leaf cuticular wax, which was the most attractive to wheat breeders. Due to the above problems, we hypothesized that cuticular wax accumulated under drought stress in wheat plants. And thus it can reduce the leaf water potential decrease, which is essential to keep plants having relatively high photosynthesis rate and relative high yield under drought stress. Therefore, it was necessary to assess the effects of leaf cuticular wax on wheat drought tolerance in an attempt to develop drought resistance cultivars.

Four wheat NILs with and without leaf cuticular wax, i.e., G-JM205, G-JM208, NG-JM204, and NG-JM206, all derived from the cross Gaocheng 9411/ATHLET, have been previously developed at our laboratory. The aims of this study are to study the effects of leaf cuticular wax on wheat grain yield under drought stress using the above-mentioned wheat NILs and to discuss the possible physiological mechanism of cuticular wax on high grain yield under drought stress.

## Materials and methods

### Plant materials and stress treatments

An F_6_ plant, derived from the cross Gaocheng 9411 (glaucous)/ATHLET (non-glaucous) and designated G-JM200, segregating in leaf bloom characteristics, was identified and confirmed by F_6:7_ families. Four wheat NILs, i.e., G-JM205, G-JM208, NG-JM204, and NG-JM206, were developed from the progenies of the above F_6_ plant and used in the current study.

Each year from 2013 to 2015, around October 10, the above-mentioned wheat NILs were sown in a randomized block experiment of three replicates with two treatments, i.e., water-irrigated (WI) and drought-stressed (DS), at the Experimental Station of Shandong Academy of Agricultural Sciences (SAAS), Jinan, Shandong Province, China. The soil contained 12.8 g·kg^−1^ of organic matter, 1.08 g·kg^−1^ of total nitrogen, 90.2 mg·kg^−1^ of alkali-nitrogen, 25.0 mg·kg^−1^ of rapidly-available phosphorus and 158.0 mg·kg^−1^ of rapidly-available potassium. The station was in a temperate continental monsoon climate, characterized by dry, cold winters and rainy, hot summers. Weather data over 2 years in 2013–2015 were recorded at a meteorological station located at the experimental site (Supplementary Table 1). During wheat growing season, total precipitation was 234.4 mm in 2013–2014 and 264.7 mm in 2014–2015. Averaged temperatures in 2013–2014 and 2014–2015 growing season were 11.7 and 11.5°C, respectively, as compared to the 30-year average of 11.2°C. Each plot measured 12 m^2^. Soil fertility was high and weeds and diseases were controlled. Under WI conditions, the wheat NILs were irrigated both at jointing and after anthesis. For the DS condition, the wheat NILs were only irrigated at jointing.

### Wax extraction

The method for cuticular wax isolation was performed as described previously by Koch et al. (2006), with minor modifications. To study the effect of drought on cuticular wax accumulation, five flag leaves were collected for four wheat NILs from the 2013–2014 field plots. The flag leaves were weighed immediately after sampling (fresh weight). Wax was extracted by dipping the flag leaf blades in 30 ml CHCl_3_ for 30 s. The wax extract was filtered using filter paper and air-dried in a desiccator at room temperature until there was no change in weight. Subsequently, the flag leaves were oven-dried for 24 h at 70°C as described by Clarke (1987). Yield of cuticular wax and dry weight (DW) of flag leaves DW were determined on an analytical balance with an accuracy of 0.01 mg (Sartorius Quintix1102-1CN, Germany). Cuticular wax content (CWC) was calculated using the following formula: CWC (mg/g) = Extracted wax weight/DW.

### Microscopic observation

Scanning Electron Microscope (SEM) imaging of cuticle surfaces was performed as previously described by Zhang et al. (2013). The detailed procedures were as followed: (1) A 0.5 cm tissue fragment was harvested from the flag leaf, the uppermost part of the flag leaf were collected from wheat spikes at anthesis at Fakes' stage 10.5.1; (2) The tissue samples were vacuum drying; (3) The pretreated samples were sputtered with gold powder using the CrC-150 Sputtering System, and inspected and captured with an SEM (SJM-6610LV, Japan).

### Measurements of flag leaf water potential (Ψ_flag leaf_), excised leaf water loss rate (ELWL), and relative water content of flag leaf (RWC)

In 2014, Ψ_flagleaf_ was measured as described by Nar et al. (2009) using a thermocouple psychrometer (Wescor PSYPRO, Logan, UT, USA). The detailed procedures were as followed: (1) Discs about 6 mm in diameter were cut from the fully expanded flag leaves at Fakes' stage 10.5.1; (2) Samples were equilibrated for 120 min before the readings were recorded by a Wescor PSYPRO water potential datalogger in the psychrometric mode; (3) Measurements were done three times from 5 leaves at the same age.

For ELWL, in order to minimize water loss due to transpiration, the excised leaves were placed in a polythene bag and then immediately transported to the laboratory. Flag leaves were excised from plants at stage 10.5.1, dehydrated for 8 h at room temperature in a dark container with a relative humidity of 40%, and weighed every 2 h using an analytical balance with an accuracy of 0.001 mg (Sartorius BSA223S, Germany). Flag leaves were then oven-dried for 24 h at 70°C as described by Clarke (1987). ELWL was calculated based on the formula: ELWL (%) = [(FW-W_*t*_)/(FW-DW)] × 100, where W_*t*_ was the weight of flag leaf after dehydration; *t* = 2, 4, 6, and 8 h, respectively. FW was the fresh weight of flag leaves. DW was the DW of flag leaves.

For relative RWC, the samples were handled as described by Dhanda and Sethi (1998), with minor modifications. The detailed procedures were as followed: (1) The samples were weighted immediately as fresh weight (FW), then sliced into 2 cm sections and floated on distilled water for 5 h; (2) The turgid leaf sections were then rapidly blotted to remove surface water and weighted to obtain turgid weight (TW); (3) The leaf discs were dried in the oven at 70°C for 24 h and then DW were obtained; (4) The relative RWC was calculated based on the formula: Relative RWC (%) = RW DS/RW WI × 100, where FW was the fresh weight of flag leaves, TW was the TW of leaf sections, and DW was the DW of flag leaf sections.

### Measurements of photosynthetic gas exchange parameters and chlorophyll (Chl) α fluorescence transient

In 2014, at the wheat flowering stage, measurements of flag leaf photosynthesis rate (*P*_*n*_, μmol·m^−2^·s^−1^), transpiration rate (*E*, mmol·m^−2^·s^−1^), stomatal conductance (*G*_*s*_, mmol·m^−2^·s^−1^), and intercellular CO_2_ concentration (*C*_*i*_, μmol·m^−2^·s^−1^) were performed as previously described by Fischer et al. (1998), with minor modifications, using a portable infrared gas analyzer (CIRAS-2 PP Systems, England) at two positions in each plot. For each measurement, which took about 30 s, the flag leaf was exposed to full sunlight, with the cuvette positioned normal to the sun to give a light intensity of 1800–2000 μmol·m^−2^·s^−1^ and three leaves were placed across the cuvette with abaxial surface of the leaves uppermost.

Also in 2014, at the flowering stage, six dark-adapted leaves were used to measure PS II activity in each plot in the dark under WI and DS conditions, respectively. The maximum PS II photochemical efficiency (*F*_*v*_*/F*_*m*_, *F*′_*v*_*/F*′_*m*_), the efficiency of electron moves beyond Q_*A*_ (ψ_*o*_, ψ′_*o*_), performance index (*PI*_*ABS*_, *PI*′_*ABS*_), and the density of Q_*A*_-reducing PS II reaction centers per cross-section (*ABS/RC, ABS*′*/RC*′) were measured using a Plant Efficiency Analyzer (PEA; Hansatech, England); measurements were carried out according to the methods described by Strasser et al. (2010).

(1) $$\begin{array}{l} F_{v}/F_{m}=1-F_{o}/F_{m},F_{v}^{\prime }/F_{m}^{\prime }=1-F_{o}^{\prime }/F_{m}^{\prime };\\ V_{j}=(F_{j}-F_{o})/(F_{m}-F_{o}), \end{array}$$

(2) $$V_{j}^{\prime }=(F_{j}^{\prime }-F_{o}^{\prime })/(F_{m}^{\prime }-F_{o}^{\prime });$$

(3) $$\psi_{o}=1-V_{j},\psi_{o}^{\prime }=1-V_{j}^{\prime };$$

(4) $$\begin{array}{l} M_{o}=4(F_{300us}-F_{o})/(F_{m}-F_{o}),\\ M_{o}^{\prime }=4(F_{300us}^{\prime }-F_{o}^{\prime })/(F_{m}^{\prime }-F_{o}^{\prime }); \end{array}$$

(5) $$\begin{array}{l} ABS/RC=M_{o}\cdot (1/V_{j})\cdot (F_{m}/F_{v}),\\ ABS^{\prime }/RC^{\prime }=M_{o}^{\prime }\cdot (1/V_{j}^{\prime })\cdot (F_{m}^{\prime }/F_{v}^{\prime }); \end{array}$$

(6) $$\begin{array}{l} PI_{ABS}=(RC/ABS)\cdot (F_{v}/F_{m})/(1-F_{v}/F_{m})\cdot \psi_{o}/(1-\psi_{o}),\\ PI_{ABS}^{\prime }=(RC^{\prime }/ABS^{\prime })\cdot (F_{v}^{\prime }/F_{m}^{\prime })/(1-F_{v}^{\prime }/F_{m}^{\prime })\cdot \psi_{o}^{\prime }/(1-\psi_{o}^{\prime }). \end{array}$$

To evaluate the effect of cuticular wax on PS II activity, the decrease of PS II activity (Δ*F*_*v*_*/F*_*m*_, Δ*ABS/RC*, Δ*PI*) induced by drought stress was calculated from the following formula: (1) Δ*F*_*v*_*/F*_*m*_ (%) = (*F*_*v*_*/F*_*m*_ – *F*_*v*_′*/F*_*m*_′)/(*F*_*v*_*/F*_*m*_) × 100; (2) Δ*ABS/RC* (%) = (*ABS/RC* – *ABS*′*/RC*′)/(*ABS/RC*) × 100; (3) Δ*PI*_*ABS*_ (%) = (*PI*_*ABS*_ – *PI'*_*ABS*_)/(*PI*_*ABS*_) × 100.

### Drought tolerance indices

Total DW was measured by harvesting 12 m^2^ of the central part of each plot at crop maturity in 2014 and 2015. Drought tolerance indices were calculated using the following relationships:

$$\text{SSI}=(1-Y_{si}/Y_{pi})/(1-Y_{s}/Y_{p})$$

(Fischer and Maurer, 1978; Mardeh et al., 2006);

TOL (kg · ha^−1^) = *Y_pi_* − *Y_si_* (Hossain et al., 1990)

where *Y*_*si*_ is the yield of cultivar under stress, *Y*_*pi*_ the yield of cultivar under irrigated condition, and *Y*_*s*_ and *Y*_*p*_ the mean yields of all cultivars under stress and non-stress conditions, respectively.

### Statistical analysis

All data was analyzed using SAS software version 9.0. A comparison was made between the mean values of wheat NILs. Duncan's multiple range test was used to test for significant differences. Statistical significance was determined at the 5% (*P* = 0.05) level.

## Results

### Wheat flag leaf cuticular wax content of wax NILs

**Figure 1:**
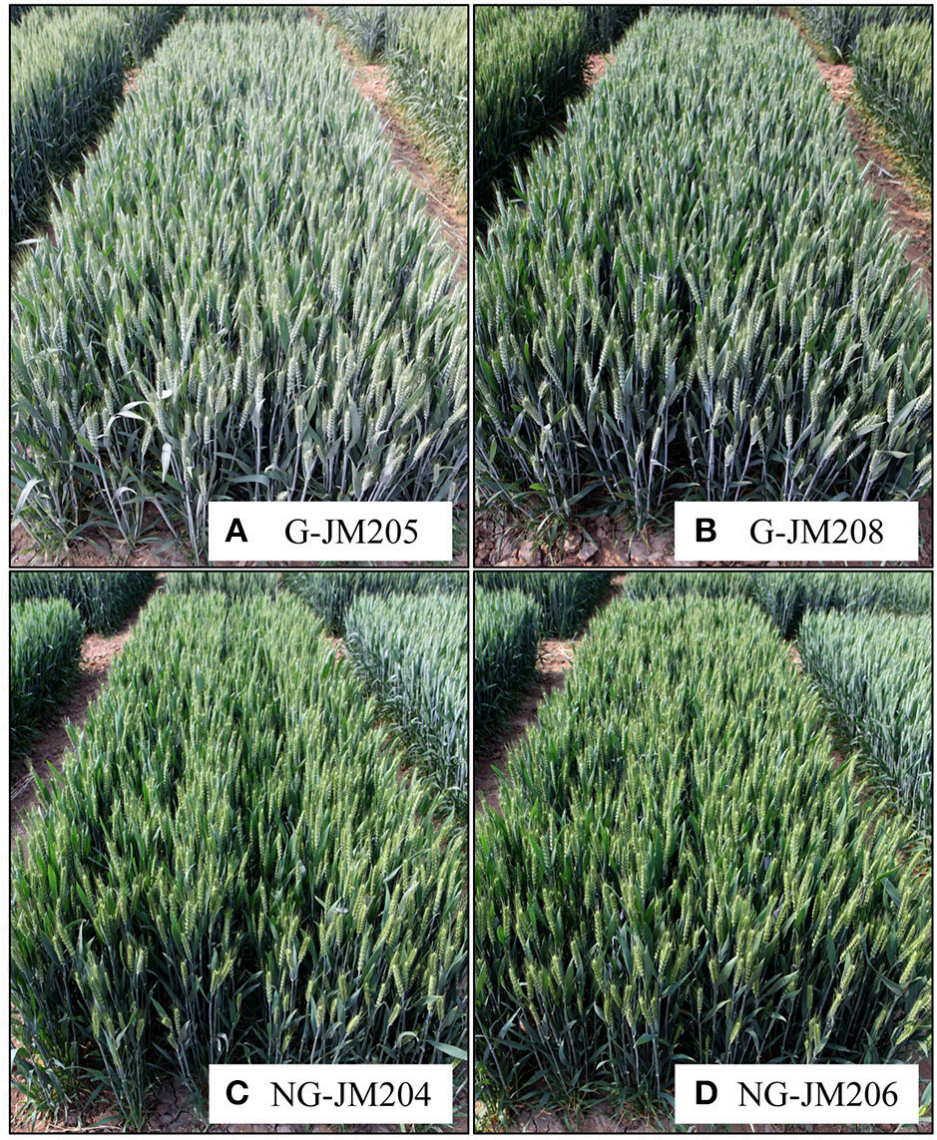
**Visual differences of wheat NILs differing in glaucousness in the field. (A)** G-JM205; **(B)** G-JM208; **(C)** NG-JM204; **(D)** NG-JM206.

Four wheat NILs, i.e., G-JM205, G-JM208, NG-JM204, and NG-JM206, were developed from an F_6_ plant, derived from the cross Gaocheng 9411 (glaucous)/ATHLET (non-glaucous); their genotypes and glaucousness of the flag leaf are shown in Figures 1A–D. The NILs G-JM205 and G-JM208 are glaucous and NG-JM204 and NG-JM206 are non-glaucous. The flag leaf CWC of wheat NILs, i.e., G-JM205, G-JM208, NG-JM204, and NG-JM206, were significantly different (*P* = 0.05), both under WI and DS conditions. In addition, the average CWC of glaucous NILs (G-JM205 and G-JM208) were significantly higher (*P* = 0.05) than that of non-glaucous NILs (NG-JM204 and NG-JM206), both under WI and DS conditions; these were a mean CWC of 43.32 mg/g and 92.88 mg/g, and 27.30 mg/g and 68.56 mg/g, respectively (Table 1). The mean CWC of the four wheat NILs under DS conditions (80.72 mg/g) was much higher than that of the NILs under WI conditions (35.31 mg/g; Table 1).

**Table 1:** **Evaluation of flag leaf CWC, Ψ_flag leaf_ and relative RWC of wheat NILs with and without cuticular wax, i.e., NG-JM204 and NG-JM206 (Bold), G-JM205 and G-JM208 (Italic) in 2014**.

**Line**	**CWC (mg/g)^a^**		**Ψflag leaf (-Mpa)**		**Relative RWC (%)**
	**Water-irrigated**	**Drought-stress**	**Water-irrigated**	**Drought-stress**	
**NG-JM204**	**26.19^c^**	**63.24^b^**	**−1.54^c^**	**−1.86^d^**	**94.66^b^**
**NG-JM206**	**28.40^c^**	**73.88^b^**	**−1.35^bc^**	**−1.60^c^**	**94.04^c^**
Average	27.30	68.56	−1.45	−1.73	94.35
*G-JM205*	*47.65*^a^	*98.91*^a^	*−0.96*^a^	*−1.15*^a^	*95.79*^a^
*G-JM208*	*39.00*^b^	*86.85*^a^	*−1.19*^b^	*−1.40*^b^	*94.70*^b^
Average	43.33	92.88	−1.07	−1.28	95.25

a *Different letters indicate significant difference among lines at P = 0.05*.

### Wax morphology

**Figure 2:**
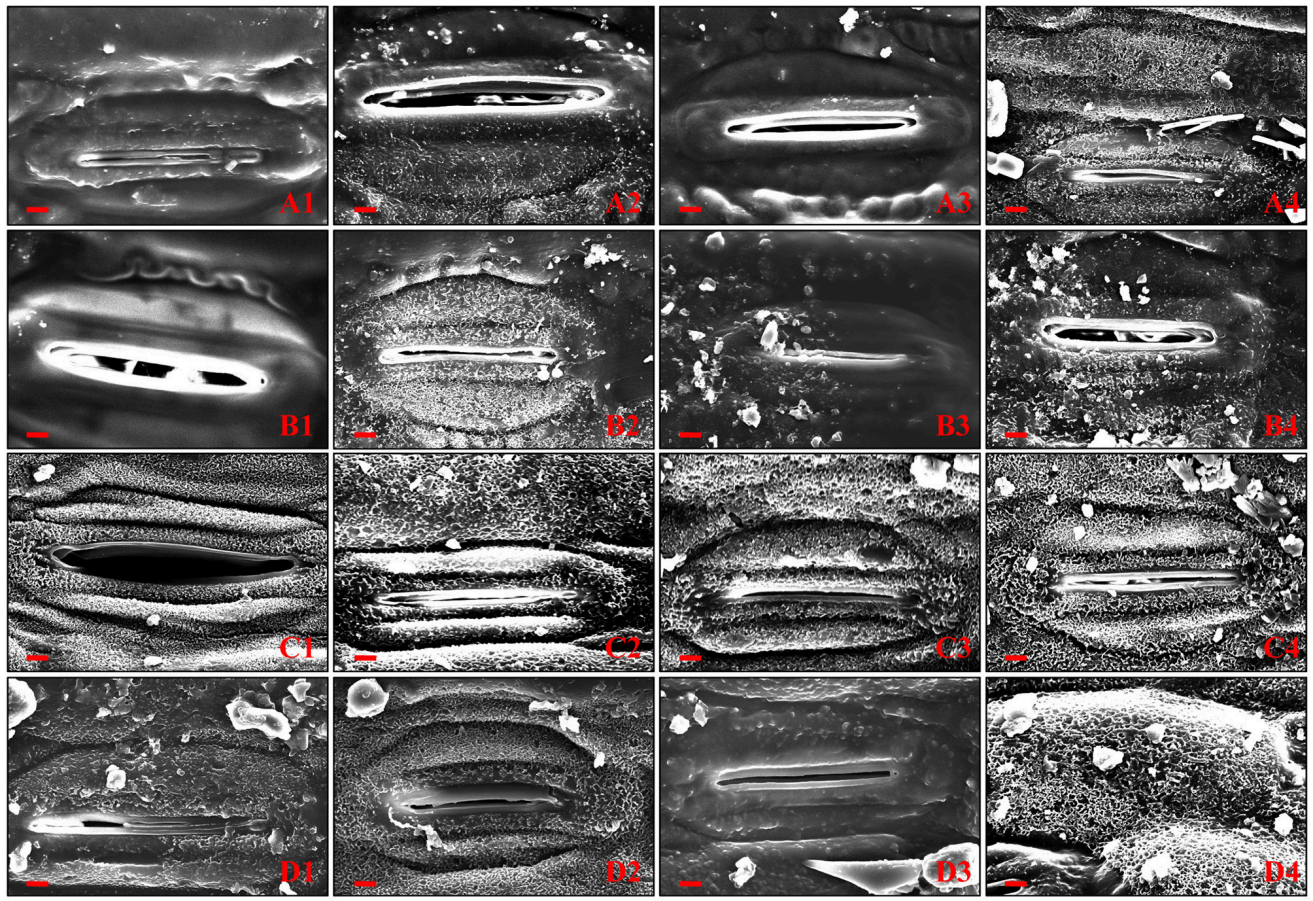
**Scanning electronic microscopy of exposed abaxial and adaxial flag leaf surfaces of non-glaucous NILs (NG-JM204 and NG-JM206) and glaucous NILs (G-JM205 and G-JM208) under WI and DS conditions**. **(A1–A4)** The abaxial flag leaf surfaces of NG-JM204, G-JM205, NG-JM206, and G-JM208 under WI conditions, respectively. **(B1–B4)** The abaxial flag leaf surfaces of NG-JM204, G-JM205, NG-JM206, and G-JM208 under DS conditions, respectively. **(C1–C4)** The adaxial flag leaf surfaces of NG-JM204, G-JM205, NG-JM206, and G-JM208 under WI conditions, respectively. **(D1–D4)** The adaxial flag leaf surfaces of NG-JM204, G-JM205, NG-JM206, and G-JM208 under DS conditions, respectively.

To determine whether the surface structure was correlated with visible phenotypes, SEM was used to examine the wax crystallites deposited on both sides of flag leaf surfaces of four wheat NIL plants (Figures 2A1–A4,C1–C4). The flag leaves of glaucous NILs (G-JM205 and G-JM208) showed a dense accumulation of tubular or rod-shaped wax structures on each surface under both WI and DS conditions (Figures 2A2,A4,B2,B4,C2,C4,D2,D4), whereas the wax crystallites deposited on the abaxial and adaxial sides of non-glaucous flag leaf surfaces were quite different (Figures 2A1,A3,B1,B3,C1,C3,D1,D3). The flag leaves of non-glaucous NILs (NG-JM204 and NG-JM206) were almost devoid of any visible wax protruding from the abaxial surface under both WI and DS conditions (Figures 2A1,A3,B1,B3), while the flag leaves of non-glaucous NILs had a dense accumulation of wax on the adaxial surfaces (Figures 2C1,C3,D1,D3). Furthermore, wax particles deposited on the abaxial surface of glaucous NILs were much denser than those on the opposite surface under both WI and DS conditions (Figures 2A1–A4,B1–B4,C1–C4,D1–D4). Moreover, compared to the flag leaf cuticular wax accumulations on both surfaces of glaucous and non-glaucous NILs under WI conditions, the wax accumulations of the four wheat NILs were much denser under DS conditions (Figures 2A1–A4,B1–B4,C1–C4,D1–D4).

### Flag leaf water potential, excised leaf water loss rate, and relative water content

**Figure 3:**
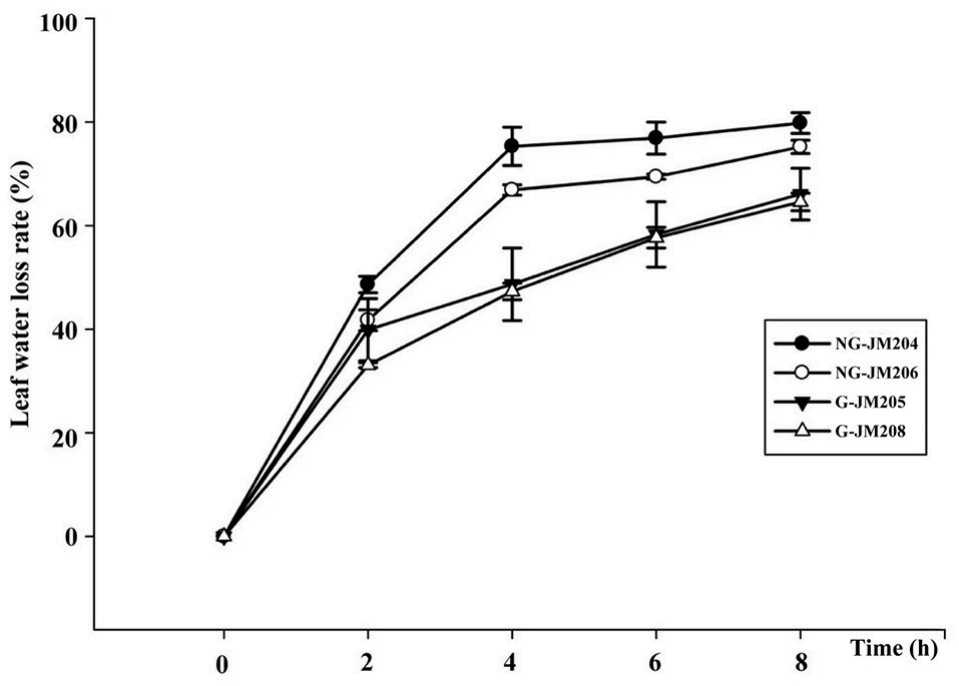
**Excised leaf water loss rate at room temperature of wheat NILs differing in glaucousness**.

The effects of flag leaf cuticular wax on physiological traits were determined by measuring Ψ_flagleaf_, ELWL, and RWC. The results indicated that under WI conditions the average Ψ_flagleaf_ of glaucous NILs was 34.4% higher than that of non-glaucous NILs, with a mean Ψ_flagleaf_ of −1.07 Mpa and −1.45 Mpa, respectively. This was also true for Ψ_flagleaf_ of glaucous and non-glaucous NILs under DS conditions, with a mean Ψ_flagleaf_ of −1.28 Mpa and −1.73 Mpa, respectively (Table 1). The effects of cuticular wax on ELWL and RWC were also measured and the results indicated that the mean RWC of glaucous NILs was higher than that of non-glaucous NILs, with a mean RWC of 95.25 and 94.35%, respectively (Table 1). Compared to non-glaucous NILs, glaucous NILs showed a lower ELWL 2 h after dehydration (*P* = 0.05) and the differences remained and increased thereafter (Figure 3). These results also indicated that the differences of Ψ_flagleaf_, ELWL, and RWC were associated with flag leaf cuticular wax.

### Wheat grain yield and drought tolerance indices

Under WI conditions, glaucous NILs (G-JM205 and G-JM208) showed a lower mean grain yield than non-glaucous NILs (NG-JM204 and NG-JM206), with mean values of 8893.62 kg·ha^−1^ and 9252.96 kg·ha^−1^ in 2014, and 7020.32 kg·ha^−1^ and 7342.54 kg·ha^−1^ in 2015, respectively. When compared to WI conditions, the mean yield of both glaucous and non-glaucous NILs decreased under DS conditions, whereas the glaucous NILs had a higher mean yield in the 2 years than the non-glaucous NILs (Table 2). Furthermore, the drought indices TOL and SSI of glaucous NILs were much lower than those of non-glaucous NILs in both years, with mean values of 588.6 kg·ha^−1^ and 0.67, and 1194.25 kg·ha^−1^ and 1.32 (Table 2), which indicated that these differences between glaucous and non-glaucous NILs were also associated with leaf cuticular wax.

**Table 2:** **Evaluation of yield and drought indices of four wheat NILs, NG-JM204, and NG-JM206 (Bold), G-JM205 and G-JM208 (Italic) in 2014 and 2015**.

**Line**	Grain yield (kg·ha^−1^)^*^		**Drought tolerance index**	**Drought susceptible index**
	**Water-irrigated**	**Drought-stress**		
**IN 2014**				
**NG-JM204**	**9096.21^b^**	**8156.71^c^**	**939.50^b^**	**1.07^a^**
**NG-JM206**	**9409.70^a^**	**8221.71^b^**	**1187.99^a^**	**1.31^a^**
Average	9252.96	8189.21	1063.75	1.19
*G-JM205*	*8854.43*^c^	*8088.37*^cd^	*766.06*^c^	*0.90*^b^
*G-JM208*	*8932.80*^c^	*8330.14*^a^	*602.66*^d^	*0.70*^b^
Average	8893.62	8209.26	684.36	0.80
**IN 2015**				
**NG-JM204**	**7856.98**^a^	**6555.22**^a^	**1301.76**^a^	**1.25**^b^
**NG-JM206**	**6828.09**^c^	**5480.40**^b^	**1347.69**^a^	**1.49**^a^
Average	7342.54	6017.81	1324.73	1.37
*G-JM205*	*7426.43^*b*^*	*6540.77^*a*^*	*885.66^*b*^*	*0.90^*c*^*
*G-JM208*	*6614.20^*cd*^*	*6355.96^*a*^*	*258.24^*c*^*	*0.30^*d*^*
Average	7020.32	6448.37	571.95	0.60

* *Different letters after the data indicate significant difference among lines at P = 0.05*.

### Flag leaf photosynthesis and chlorophyll fluorescence

**Figure 4:**
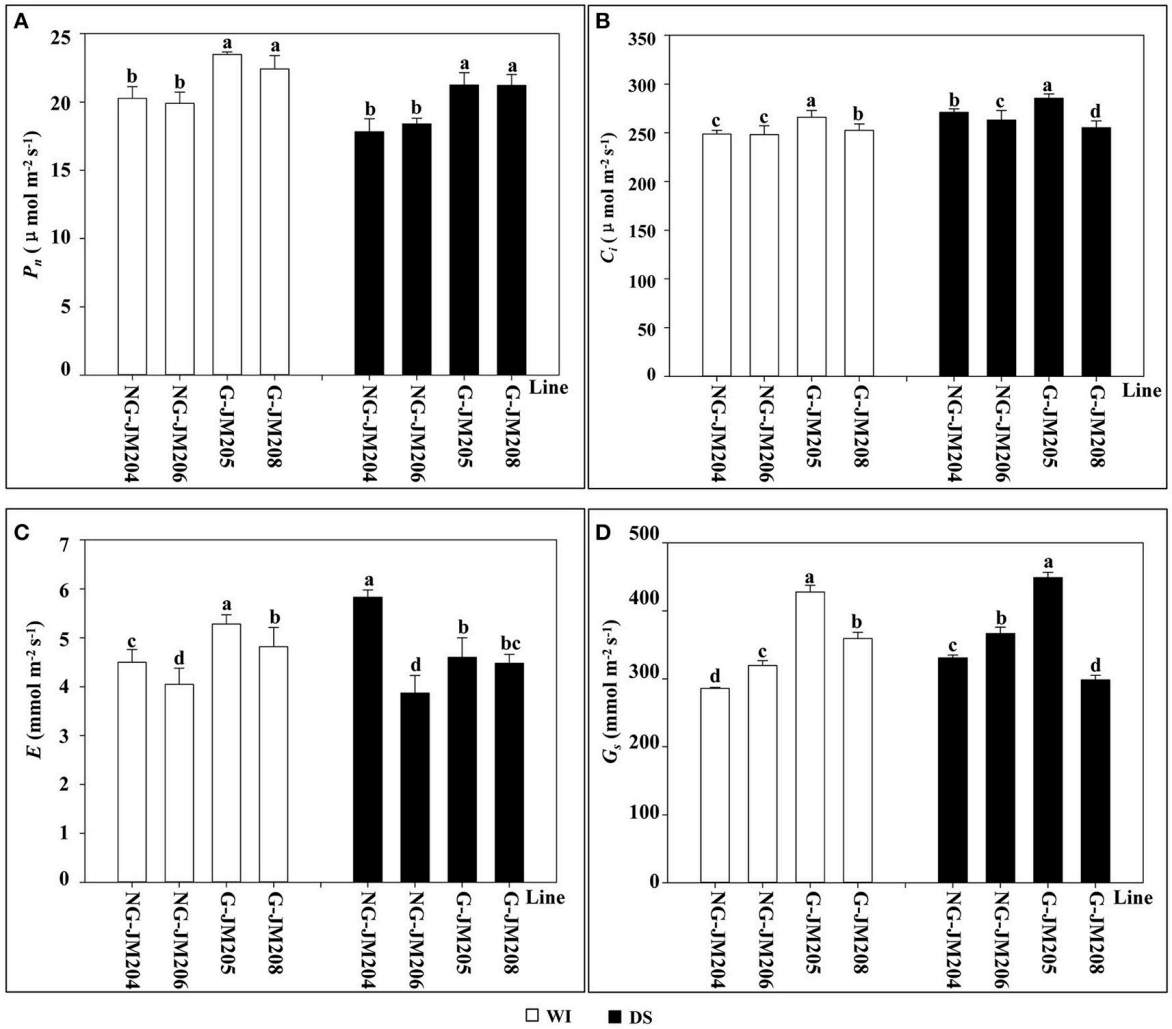
**Comparison of flag leaf photosynthetic rate *P*_*n*_ (A)**, intercellular CO_2_ concentration *C*_*i*_ **(B)**, transpiration rate *E* **(C)** and stomatal conductance *G*_*s*_ **(D)** between wheat NILs under WI and DS conditions. Panels **A–D**: Column diagrams with *white* and *black* color represents the changes of flag leaf photosynthetic rate *P*_*n*_, intercellular CO_2_ concentration *C*_*i*_, transpiration rate *E* and stomatal conductance *G*_*s*_ in wheat NILs, NG-JM204, NG-JM206, G-JM205 and G-JM208 under WI and DS conditions, respectively. Different letters above the column diagrams indicate significant difference among lines at *P* = 0.05.

Under WI conditions, glaucous NILs (G-JM205 and G-JM208) showed higher mean values of *P*_*n*_, *G*_*s*_, *E*, and *C*_*i*_ than those of non-glaucous NILs (NG-JM204 and NG-JM206; *P* = 0.05). Compared to WI conditions, the *P*_*n*_ of wheat glaucous and non-glaucous NILs under DS conditions decreased 7.5 and 9.8%, respectively; however, glaucous NILs still had higher mean values of *P*_*n*_ than those of non-glaucous NILs (Figure 4A). In addition, compared to WI conditions, the average *G*_*s*_ and *E* of glaucous NILs under DS conditions decreased 5.1 and 10.1%, respectively, while the mean values of *G*_*s*_ and *E* in non-glaucous NILs increased 14.9 and 13.5%, respectively (Figures 4C,D), indicating that these differences were also associated with leaf cuticular wax. However, compared to WI conditions, the *C*_*i*_ of both glaucous and non-glaucous NILs under DS conditions increased 4.3 and 7.5%, respectively (Figure 4B).

To evaluate the effect of cuticular wax on PS II activity, the maximum PS II photochemical efficiency (*F*_*v*_*/F*_*m*_, *F*′_*v*_*/F*′_*m*_), the efficiency of electron moves beyond Q_A_ (ψ_*o*_, ψ′_*o*_), performance index (*PI, PI*′), and the density of Q_*A*_-reducing PS II reaction centers per cross-section (*ABS/RC, ABS'/RC'*) were measured using a Plant Efficiency Analyzer (PEA, Hansatech, England) in the dark under WI and DS conditions, respectively. Subsequently, the decrease of PS II activity (Δ*F*_*v*_*/F*_*m*_, Δ*ABS/RC*, Δ*PI*) induced by strong light was calculated. Compared to the chlorophyll fluorescence indices measured in the dark under WI conditions, the maximum PS II photochemical efficiency (*F*_*v*_*/F*_*m*_) and the performance index (*PI*) of glaucous NILs (G-JM205 and G-JM208) and non-glaucous NILs (NG-JM204 and NG-JM206) under DS conditions measured in the dark both decreased, with mean values of 4.6% and 5.6%, and 39.5% and 43.0% (Table 3; Supplementary Table 2). While the density of *Q*_*A*_-reducing PS II reaction centers per cross-section (*ABS/RC*) of glaucous NILs and non-glaucous NILs under DS conditions increased, with mean values of 42.4 and 37.5%, which reflected the photosynthesis data.

**Table 3:** **The decrease of PS II activity in four NILs, NG-JM204 and NG-JM206 (Bold), G-JM205 and G-JM208 (Italic) under DS conditions in 2014**.

**Line**	ΔF_*v*_/F_m_^*^	ΔABS/RC^*^	ΔPI^*^
**NG-JM204**	**−5.6^a^**	**34.2^c^**	**−44.6^a^**
**NG-JM206**	**−5.6^a^**	**40.8^b^**	**−41.5^b^**
Average	−5.6	37.5	−43.0
*G-JM205*	*−5.1^b^*	*41.9^a^*	*−41.6^b^*
*G-JM208*	*−4.0^c^*	*42.9^a^*	*−37.5^c^*
Average	−4.6	42.4	−39.5

* *Letters after the data indicate significant difference at P = 0.05. “−” and “+”before the data represent increase and decrease, respectively*.

## Discussion

Drought is a major factor that seriously restricts wheat production (Fleury et al., 2010; Piao et al., 2010). Previous studies have indicated that leaf cuticular wax is associated with plant drought tolerance in many species, such as *Arabidopsis*, cotton and wheat (Bondada et al., 1996; Kosma et al., 2009; Zhang et al., 2015). And Chatterton et al. (1975) reported that sorghum yield was associated with cuticular wax; however, wheat drought tolerance conferred by cuticular wax has rarely been studied with NILs in wheat (Uddin and Marshall, 1988; Araus et al., 1991; Zhang et al., 2005; Cameron et al., 2006; Kim et al., 2007; Yang et al., 2011). In the current study, two pairs of wheat NILs with and without leaf cuticular wax, i.e., glaucous NILs (G-JM205 and G-JM208) and non-glaucous NILs (NG-JM204 and NG-JM206), were firstly utilized to study leaf cuticular wax accumulation and its relationship to wheat grain yield under drought stress. The results indicated that, when compared to WI conditions, the mean CWC of glaucous and non-glaucous NILs were both increased under DS conditions but the percentage increase in mean CWC was higher in non-glaucous NILs than in glaucous NILs (Table 1). Mean increases under DS conditions were 151.1 and 114.4%, respectively, which was corroborated by the large wax particles loaded on the surfaces of flag leaves as detected by SEM (Figure 2); this indicated that drought can induce the expression of cuticular wax biosynthesis genes in plants (Samdur et al., 2003; Zhang et al., 2005; Kosma et al., 2009; Yang et al., 2011; Zhu et al., 2014). Furthermore, under DS conditions, the mean yield of glaucous NILs was higher than that of non-glaucous NILs (*P* = 0.05). In addition, compared to WI conditions, the average yield decrease of glaucous NILs inferred by TOL was much lower than that of non-glaucous NILs, and glaucous NILs had lower values of SSI than non-glaucous NILs (*P* = 0.05; Table 2). This indicated that the leaf cuticular wax accumulation was associated with wheat drought tolerance (González and Ayerbe, 2010).

Ψ_flagleaf_, ELWL, and RWC, three important indices, have been used to measure plant drought tolerance (Centritto et al., 1999; Zhang et al., 2004, 2013; Verslues et al., 2006). In the current study, Ψ_flagleaf_, ELWL, and RWC were used to measure the drought tolerance of wheat glaucous and non-glaucous NILs. The results indicated that the ELWL of glaucous NILs 8 h after dehydration was lower than that of non-glaucous NILs (Figure 3), with mean values of 65.33 and 77.50%, respectively. This was consistent with previous studies that show that leaf cuticular wax can reduce leaf water loss through non-stomatal conductance to maintain a relative high water potential in the leaf (Zhang et al., 2013; Wang et al., 2014a). Moreover, the RWC of glaucous NILs was higher than that of non-glaucous NILs (Table 1), indicating that glaucous NILs would be more tolerant of water deficit than non-glaucous NILs (Matin et al., 1989). In addition, the mean Ψ_flagleaf_ of glaucous NILs and non-glaucous NILs under DS conditions were both lower than those under WI conditions. Under DS conditions, the glaucous NILs had higher Ψ_flagleaf_ than non-glaucous NILs (*P* = 0.05; Table 1), which indicated that drought can induce a decrease of leaf water potential and also suggested that the accumulated cuticular wax can keep plants in a relatively high water potential by reducing the leaf transpiration rate, which was positively related to plant water use efficiency and was essential for high leaf photosynthesis in wheat (Matin et al., 1989; Jongdee et al., 2002; Porcel and Ruiz-Lozano, 2004).

Indicators of PS II activity were seriously affected by various stresses, such as drought and salt (Maxwell and Johnson, 2000; Shepherd and Wynne Griffiths, 2006) and the photoinactivation of PS II complexes photoprotect functional neighbors (Anderson et al., 1997; Lee et al., 2001). In the present study, compared to the chlorophyll fluorescence indices measured in the dark under WI conditions, the *F*_*v*_*/F*_*m*_ of glaucous NILs (G-JM205 and G-JM208) and non-glaucous NILs (NG-JM204 and NG-JM206) under DS conditions measured in the dark decreased (Table 3), which indicated that photosynthetic electron transport chain was sensitive to drought stress. Cuticular wax, as a photoprotective layer, can protect plants against UV radiation and drought stress (Butler, 1996; Shepherd and Wynne Griffiths, 2006). In this study, glaucous NILs had lower *F*_*v*_*/F*_*m*_ decrease than that of non-glaucous NILs (Table 3 and Supplementary Table 1), suggesting that the plant protected its PSII against drought by accumulation of large amount of cuticular wax.

*ABS/RC*, the effective antenna size of an active reaction center (RC), is one of the four RC parameters (*ABS/RC, TR*_0_*/RC, ET*_0_*/RC*, and *DI*_0_*/RC*), which reflects the stepwise flow of energy through PS II at the RC level, which is influenced by the ratio of active/inactive RCs (Strasser and Strasser, 1995). In this study, the glaucous NILs had greater *ABS/RC* increase (42.4%) than non-glaucous NILs (37.5%), showing that drought can induce the effective antenna size increasing. And the greater increase in *ABS/RC* observed in the glaucous NILs leaves compared with the non-glaucous NILs leaves, may suggest that the leaf cuticular wax can be able to regulate the amount of light reaching the RC under drought stress.

*PI* was a very sensitive parameter in different crops and in most of environmental stress situations (Strasser et al., 2004; Christen et al., 2007; Oukarroum et al., 2007). It reflected the functionality of both PS I and II and gave us quantitative information on the current state of plant performance under stress conditions (Strasser et al., 2004; Živčák et al., 2008). In the present study, *PI* of glaucous NILs (G-JM205 and G-JM208) and non-glaucous NILs (NG-JM204 and NG-JM206) under DS conditions measured in the dark decreased. Compared to values of *F*_*v*_*/F*_*m*_, *PI* showed a much greater decrease in values, which was consistent with previous results that *PI* was more sensitive to the environmental factors than *F*_*v*_*/F*_*m*_ (Li et al., 2005; Živčák et al., 2008; Su et al., 2014; Wang et al., 2014b). On the other hand, The higher decrease in *PI* in the non-glaucous leaves than in the glaucous leaves supports the idea that cuticular wax is an important protector for photosynthesis under drought stress.

Photosynthesis is the basis for the accumulation of plant dry mass. In this study, the average *P*_*n*_ of glaucous NILs was higher than that of non-glaucous NILs under both WI and DS conditions (Figure 4A). When compared to WI conditions, the average *P*_*n*_ of both glaucous and non-glaucous NILs decreased due to drought stress, and glaucous NILs had a smaller *P*_*n*_ reduction than non-glaucous NILs under DS conditions. This indicated that plant photosynthesis was substantially affected by drought tolerance (Yordanov et al., 2000; Reddy et al., 2004) and also suggested that leaf cuticular wax can protect the flag leaf from harm caused by drought stress and maintain a relatively high *P*_*n*_, which may directly result in the higher grain yield of glaucous NILs (Table 2). On the other hand, at the late grain-filling stage, glaucous NILs had a longer stay-green stage (~ 2 days) than non-glaucous NILs, which was also a possible reason for the relatively high yield of glaucous NILs under DS conditions (Table 2). However, glaucous NILs had a higher *P*_*n*_ than non-glaucous NILs under WI conditions, which means the former would have higher biomass than the later. Concerning that wheat yield is determined by biomass and harvest index (HI), or spike numbers per unit, spikelet numbers per spike and thousand kernel weight (TKW) (Reynolds et al., 2009, 2012), further studies are likely to allow us to understand the effects of cuticular wax on yield related traits.

Plants are protected by several mechanisms capable of preventing drought-induced photodamage, the most important of which is accumulation of cuticular wax (Shepherd and Wynne Griffiths, 2006). As a result of this study, we can conclude that cuticular wax can minimize adverse effects of the high level of drought stress by reducing leaf tranpiration and maintains stomatal conductance under drought stress. And the results also confirmed that cuticular wax, as a photoprotective layer, saved PS II complex in plants under drought stress.

Plant physiological traits as efficient methods are very advantageous with a potential for use in plant screening for stress tolerance. The main problem, especially in selection for improved drought tolerance, is the lack of reliable and sufficiently sensitive parameters of selection. Our results show that cuticular wax can maintain a relatively high water potential in the flag leaf, a relatively low ELWL, and a relatively high RWC, which indirectly resulted in the relative high *P*_*n*_, PS II activity and grain yield of glaucous NILs under DS conditions. And leaf CWC is able to reflect the effect of drought stress on wheat grain yield, providing a suitable screening protocol is designed, which can also be used to differentiate tested wheat varieties to more or less drought tolerant.

## Author contributions

JS and SZ conceived and designed the experiments. WX, JG, HS, HL, and AL performed the experiments. JG, WX, XY, and JS analyzed the data. CL, DC, and JL contributed reagents/materials/analysis tools. JG and JS wrote the paper.

## Funding

This research was financially supported by the NSF of China (31271635), and partially by National Modern Agricultural Industry System Construction Project (CARS-03-1-8), Shandong Province Key Technology Innovation Project (2014GJJS0201-1), and The Scholars of Taishan Seed Industry Project (2014–2019).

### Conflict of interest statement

The authors declare that the research was conducted in the absence of any commercial or financial relationships that could be construed as a potential conflict of interest.

## References

[B1] Adamski N. M. Bush M. S. Simmonds J. Turner A. S. Mugford S. G. Jones A.. (2013). The inhibitor of wax 1 locus (Iw1) prevents formation of β- and OH-β-diketones in wheat cuticular waxes and maps to a sub-cM interval on chromosome arm 2BS. Plant J. 74, 989–1002. 10.1111/tpj.12185 23551421

[B2] Anderson J. M. Park Y. I. Chow W. S. (1997). Photoinactivation and photoprotection of photosystem II in nature. Physiol. Plantarum. 100, 214–223. 10.1111/j.1399-3054.1997.tb04777.x

[B3] Araus J. Febrero A. Vendrell P. (1991). Epidermal conductance in different parts of durum wheat grown under Mediterranean conditions: the role of epicuticular waxes and stomata. Plant Cell Environ. 14, 545–558. 10.1111/j.1365-3040.1991.tb01525.x

[B4] Arnell N. W. (2008). Climate change and drought, in Drought Management: Scientific and Technological Innovations, Options Méditerranéennes: Série A. Séminaires Méditerranéens; n. 80, ed López-Francos A. (Zaragoza: CIHEAM), 13–19. Available online at: http://om.ciheam.org/om/pdf/a80/00800414.pdf

[B5] Blum A. Ebercon A. (1981). Cell membrane stability as a measure of drought and heat tolerance in wheat. Crop Sci. 21, 43–47. 10.2135/cropsci1981.0011183X002100010013x

[B6] Bondada B. R. Oosterhuis D. M. Murphy J. B. Kim K. S. (1996). Effect of water stress on the epicuticular wax composition and ultrastructure of cotton (*Gossypium hirsutum* L.) leaf, bract, and boll. Environ. Exp. Bot. 36, 61–69. 10.1016/0098-8472(96)00128-1

[B7] Butler D. R. (1996). The presence of water on leaf surfaces and its importance for microbes and insects, in Plant Cuticles: An Integrated Functional Approach, ed Kerstiens G. (Oxford: BIOS), 267–282.

[B8] Cameron K. D. Teece M. A. Smart L. B. (2006). Increased accumulation of cuticular wax and expression of lipid transfer protein in response to periodic drying events in leaves of tree tobacco. Plant Physiol. 140, 176–183. 10.1104/pp.105.069724 16361524 PMC1326042

[B9] Centritto M. Lee H. S. Jarvis P. G. (1999). Interactive effects of elevated CO2 and drought on cherry (*Prunus avium*) seedlings I. Growth, whole-plant water use efficiency and water loss. New Phytol. 141, 129–140. 10.1046/j.1469-8137.1999.00326.x

[B10] Chatterton N. J. Lee D. Powell J. B. Hanna W. (1975). Photosynthesis and transpiration of bloom and bloomless sorghum. Can. J. Plant Sci. 55, 641–643. 10.4141/cjps75-097

[B11] Christen D. Schönmann S. Jermini M. Strasser R. J. Défago G. (2007). Characterization and early detection of grapevine (*Vitis vinifera*) stress responses to esca disease by *in situ* chlorophyll fluorescence and comparison with drought stress. Environ. Exp. Bot. 60, 504–514. 10.1016/j.envexpbot.2007.02.003

[B12] Clarke J. M. (1987). Use of physiological and morphological traits in breeding programmes to improve drought resistance of cereals, in Drought Tolerance in Winter Cereals, ed Srivastava J. P. Porcedo E. Acevedo E. Varma S. (New York, NY: John Wiley & Sons), 171–189.

[B13] Dhanda S. Sethi G. (1998). Inheritance of excised-leaf water loss and relative water content in bread wheat (*Triticum aestivum*). Euphytica 104, 39–47. 10.1023/A:1018644113378

[B14] FAO (2013). Drought. Available online at: http://www.fao.org/docrep/017/aq191e/aq191e.pdf.

[B15] Fischer R. Maurer R. (1978). Drought resistance in spring wheat cultivars. I. Grain yield responses. Crop Pasture Sci. 29, 897–912. 10.1071/AR9780897

[B16] Fischer R. Rees D. Sayre K. Lu Z. M. Condon A. Saavedra A. L. (1998). Wheat yield progress associated with higher stomatal conductance and photosynthetic rate, and cooler canopies. Crop Sci. 38, 1467–1475. 10.2135/cropsci1998.0011183X003800060011x

[B17] Fleury D. Jefferies S. Kuchel H. Langridge P. (2010). Genetic and genomic tools to improve drought tolerance in wheat. J. Exp. Bot. 61, 3211–3222. 10.1093/jxb/erq152 20525798

[B18] Foulkes M. Sylvester-Bradley R. Weightman R. Snape J. (2007). Identifying physiological traits associated with improved drought resistance in winter wheat. Field Crop. Res. 103, 11–24. 10.1016/j.fcr.2007.04.007

[B19] González A. Ayerbe L. (2010). Effect of terminal water stress on leaf epicuticular wax load, residual transpiration and grain yield in barley. Euphytica 172, 341–349. 10.1007/s10681-009-0027-0

[B20] Hossain A. Sears R. Cox T. Paulsen G. (1990). Desiccation tolerance and its relationship to assimilate partitioning in winter wheat. Crop Sci. 30, 622–627. 10.2135/cropsci1990.0011183X003000030030x

[B21] Islam M. A. Du H. Ning J. Ye H. Xiong L. (2009). Characterization of Glossy1-homologous genes in rice involved in leaf wax accumulation and drought resistance. Plant Mol. Biol. 70, 443–456. 10.1007/s11103-009-9483-0 19322663

[B22] Johnson D. A. Richards R. A. Turner N. C. (1983). Yield, water relations, gas exchange, and surface reflectances of near-isogenic wheat lines differing in glaucousness. Crop Sci. 23, 318–325. 10.2135/cropsci1983.0011183X002300020033x

[B23] Jongdee B. Fukai S. Cooper M. (2002). Leaf water potential and osmotic adjustment as physiological traits to improve drought tolerance in rice. Field Crop Res. 76, 153–163. 10.1016/S0378-4290(02)00036-9

[B24] Kim K. S. Park S. H. Jenks M. A. (2007). Changes in leaf cuticular waxes of sesame (*Sesamum indicum* L.) plants exposed to water deficit. J. Plant Physiol. 164, 1134–1143. 10.1016/j.jplph.2006.07.004 16904233

[B25] Koch K. Barthlott W. Koch S. Hommes A. Wandelt K. Mamdouh W. (2006). Structural analysis of wheat wax (*Triticum aestivum*, cv. ‘Naturastar’L.): from the molecular level to three dimensional crystals. Planta 223, 258–270. 10.1007/s00425-005-0081-3 16133211

[B26] Kosma D. K. Bourdenx B. Bernard A. Parsons E. P. Lü S. Joubès J.. (2009). The impact of water deficiency on leaf cuticle lipids of Arabidopsis. Plant Physiol. 151, 1918–1929. 10.1104/pp.109.141911 19819982 PMC2785987

[B27] Lee H. Y. Hong Y. N. Chow W. S. (2001). Photoinactivation of photosystem II complexes and photoprotection by non-functional neighbours in *Capsicum annuum* L. Leaves. Planta 212, 332–342. 10.1007/s004250000398 11289597

[B28] Li P. M. Gao H. Y. Strasser R. J. (2005). Application of the fast chlorophyll fluorescence induction dynamics analysis in photosynthesis study. Acta Photophysiol. Sin. 31, 559–566. Available online at: http://en.cnki.com.cn/Article_en/CJFDTOTAL-ZWSI200506001.htm 16361781

[B29] Li X. Waddington S. R. Dixon J. Joshi A. K. De Vicente M. C. (2011). The relative importance of drought and other water-related constraints for major food crops in South Asian farming systems. Food Secur. 3, 19–33. 10.1007/s12571-011-0111-x

[B30] Mardeh A. S. S. Ahmadi A. Poustini K. Mohammadi V. (2006). Evaluation of drought resistance indices under various environmental conditions. Field Crop Res. 98, 222–229. 10.1016/j.fcr.2006.02.001

[B31] Matin M. Brown J. H. Ferguson H. (1989). Leaf water potential, relative water content, and diffusive resistance as screening techniques for drought resistance in barley. Agron. J. 81, 100–105. 10.2134/agronj1989.00021962008100010018x

[B32] Maxwell K. Johnson G. N. (2000). Chlorophyll fluorescence-a practical guide. J. Exp. Bot. 51, 659–668. 10.1093/jexbot/51.345.659 10938857

[B33] Nar H. Saglam A. Terzi R. Varkonyi Z. Kadioglu A. (2009). Leaf rolling and photosystem II efficiency in Ctenanthe setosa exposed to drought stress. Photosynthetica 47, 429–436. 10.1007/s11099-009-0066-8

[B34] Oukarroum A. El Madidi S. Schansker G. Strasser R. J. (2007). Probing the responses of barley cultivars (*Hordeum vulgare* L.) by chlorophyll a fluorescence OLKJIP under drought stress and re-watering. Environ. Exp. Bot. 60, 438–446. 10.1016/j.envexpbot.2007.01.002

[B35] Piao S. Ciais P. Huang Y. Shen Z. Peng S. Li J.. (2010). The impacts of climate change on water resources and agriculture in China. Nature 467, 43–51. 10.1038/nature09364 20811450

[B36] Porcel R. Ruiz-Lozano J. M. (2004). Arbuscular mycorrhizal influence on leaf water potential, solute accumulation, and oxidative stress in soybean plants subjected to drought stress. J. Exp. Bot. 55, 1743–1750. 10.1093/jxb/erh188 15208335

[B37] Reddy A. R. Chaitanya K. V. Vivekanandan M. (2004). Drought-induced responses of photosynthesis and antioxidant metabolism in higher plants. J. Plant Physiol. 161, 1189–1202. 10.1016/j.jplph.2004.01.013 15602811

[B38] Reynolds M. Foulkes J. Furbank R. Griffiths S. King J. Murchie E.. (2012). Achieving yield gains in wheat. Plant Cell Environ. 35, 1799–1823. 10.1111/j.1365-3040.2012.02588.x 22860982

[B39] Reynolds M. Foulkes M. J. Slafer G. A. Berry P. Parry M. A. Snape J. W.. (2009). Raising yield potential in wheat. J. Exp. Bot. 60, 1899–1918. 10.1093/jxb/erp016 19363203

[B40] Samdur M. Manivel P. Jain V. Chikani B. Gor H. Desai S. (2003). Genotypic differences and water-deficit induced enhancement in epicuticular wax load in peanut. Crop Sci. 43, 1294–1299. 10.2135/cropsci2003.1294

[B41] Shepherd T. Wynne Griffiths D. (2006). The effects of stress on plant cuticular waxes. New Phytol. 171, 469–499. 10.1111/j.1469-8137.2006.01826.x 16866954

[B42] Strasser B. J. Strasser R. J. (1995). Measuring fast fluorescence transients to address environmental questions: The JIP-test, in Photosynthesis: From Light to Biosphere, ed Mathis P. (Dordrecht; Kluwer Academic Publishers), 977–980.

[B43] Strasser R. J. Tsimilli-Michael M. Qiang S. Goltsev V. (2010). Simultaneous *in vivo* recording of prompt and delayed fluorescence and 820-nm reflection changes during drying and after rehydration of the resurrection plant *Haberlea rhodopensis*. Biochim. Biophys. Acta 1797, 1313–1326. 10.1016/j.bbabio.2010.03.008 20226756

[B44] Strasser R. J. Tsimilli-Michael M. Srivastava A. (2004). Analysis of the chlorophyll a fluorescence transient, in Chlorophyll a Fluorescence, ed Papageorgiou G. C. Govindjee (Netherlands; Springer), 321–362. 10.1007/978-1-4020-3218-9_12

[B45] Su X. Wu S. Yang L. Xue R. Li H. Wang Y. (2014). Exogenous progesterone alleviates heat and high light stress-induced inactivation of photosystem II in wheat by enhancing antioxidant defense and D1 protein stability. Plant Growth Regul. 74, 311–318. 10.1007/s10725-014-9920-1

[B46] Tsunewaki K. Ebana K. (1999). Production of near-isogenic lines of common wheat for glaucousness and genetic basis of this trait clarified by their use. Genes Genet. Syst. 74, 33–41. 10.1266/ggs.74.33

[B47] Uddin M. N. Marshall D. (1988). Variation in epicuticular wax content in wheat. Euphytica 38, 3–9. 10.1007/BF00024805

[B48] Verslues P. E. Agarwal M. Katiyar-Agarwal S. Zhu J. Zhu J. K. (2006). Methods and concepts in quantifying resistance to drought, salt and freezing, abiotic stresses that affect plant water status. Plant J. 45, 523–539. 10.1111/j.1365-313X.2005.02593.x 16441347

[B49] Wang J. Li W. Wang W. (2014a). Fine mapping and metabolic and physiological characterization of the glume glaucousness inhibitor locus Iw3 derived from wild wheat. Theor. Appl. Genet. 127, 831–841. 10.1007/s00122-014-2260-8 24522723

[B50] Wang Y. Zhang H. Hou P. Su X. Zhao P. Zhao H. (2014b). Foliar-applied salicylic acid alleviates heat and high light stress induced photoinhibition in wheat (*Triticum aestivum*) during the grain filling stage by modulating the psbA gene transcription and antioxidant defense. Plant Growth Regul. 73, 289–297. 10.1007/s10725-014-9889-9

[B51] Wójcicka A. (2015). Surface waxes as a plant defense barrier towards grain aphid. Acta Biol. Cracov. Bot. 57, 95–103. 10.1515/abcsb-2015-0012

[B52] Wu H. Qin J. Han J. Zhao X. Ouyang S. Liang Y.. (2013). Comparative high-resolution mapping of the wax inhibitors Iw1 and Iw2 in hexaploid wheat. PLoS ONE 8:e84691. 10.1371/journal.pone.0084691 24376835 PMC3871689

[B53] Yang J. Ordiz M. I. Jaworski J. G. Beachy R. N. (2011). Induced accumulation of cuticular waxes enhances drought tolerance in Arabidopsis by changes in development of stomata. Plant Physiol. Biochem. 49, 1448–1455. 10.1016/j.plaphy.2011.09.006 22078383

[B54] Yordanov I. Velikova V. Tsonev T. (2000). Plant responses to drought, acclimation, and stress tolerance. Photosynthetica 38, 171–186. 10.1023/A:1007201411474

[B55] Zhang J. Y. Broeckling C. D. Blancaflor E. B. Sledge M. K. Sumner L. W. Wang Z. Y. (2005). Overexpression of WXP1, a putative *Medicago truncatula* AP2 domain-containing transcription factor gene, increases cuticular wax accumulation and enhances drought tolerance in transgenic alfalfa (*Medicago sativa*). Plant J. 42, 689–707. 10.1111/j.1365-313X.2005.02405.x 15918883

[B56] Zhang M. Peng Z. Xie B. Tan F. Yang Y. (2004). Relationship between water loss rate of cutting leaves and osmotic regulators under water stress and drought resistance in sweet potato. Sci. Agric. Sin. 37, 152–156. Available online at: http://en.cnki.com.cn/Article_en/CJFDTotal-ZNYK200401024.htm

[B57] Zhang Z. Wang W. Li W. (2013). Genetic interactions underlying the biosynthesis and inhibition of β-diketones in wheat and their impact on glaucousness and cuticle permeability. PLoS ONE 8:e54129. 10.1371/journal.pone.0054129 23349804 PMC3547958

[B58] Zhang Z. Wei W. Zhu H. Challa G. S. Bi C. Trick H. N.. (2015). W3 is a new wax locus that is essential for biosynthesis of β-Diketone, development of glaucousness, and reduction of cuticle permeability in common wheat. PLoS ONE 10:e0140524. 10.1371/journal.pone.0140524 26468648 PMC4607432

[B59] Zhu L. Guo J. Zhu J. Zhou C. (2014). Enhanced expression of EsWAX1 improves drought tolerance with increased accumulation of cuticular wax and ascorbic acid in transgenic Arabidopsis. Plant Physiol. Biochem. 75, 24–35. 10.1016/j.plaphy.2013.11.028 24361507

[B60] Živčák M. Brestič M. Olšovská K. Slamka P. (2008). Performance index as a sensitive indicator of water stress in *Triticum aestivum* L. Plant Soil Environ. 54, 133–139. Available online at: http://agriculturejournals.cz/publicFiles/01163.pdf

