# Supplementary material for: Cuticular Wax Accumulation Is Associated with Drought Tolerance in Wheat Near-Isogenic Lines
Source: Front Plant Sci. 2016 Nov 30;7:1809. doi: 10.3389/fpls.2016.01809 (PMC5129171; doi:10.3389/fpls.2016.01809)
Supplement: Supplementary file 1 [file Presentation1.PDF]

Supplementary Material

Assessing the effect of cuticular wax on drought tolerance using wheat near isogenic lines

Jun Guo[1], Wen Xu[2**], Xiaocong Yu[1**], Hao Shen[2], Haosheng Li[1], Dungong Cheng[1], Aifeng Liu[1], Jianjun Liu[1], Cheng Liu[1], Shijie Zhao[2*], Jianmin Song[1*]**

[1]Crop Research Institute, National Engineering Laboratory for Wheat and Maize & Key Laboratory of Wheat Biology and Genetic Improvement in North Yellow and Huai River Valley, Ministry of Agriculture, Shandong Academy of Agricultural Sciences (SAAS), No. 202, Gongye North Road, Jinan 250100, China

[2]College of Life Sciences, State Key Laboratory of Crop Biology, Shandong Agricultural University (SDAU), Taian 271018, China

[**]Authors contributed equally to this work.

[*]Correspondence: Corresponding author: J Song, Tel: +86-531-8317 9561, e-mail: wheat_breeding2016@163.com; S Zhao, Tel:+86-538-824-9767, e-mail: sjzhao@sdau.edu.cn

Supplementary Material

1 Supplementary Tables

1.1 Supplementary Table 1 Monthly average air temperature, precipitation, and sunshine hours in the 2013-2014 and 2014-2015 growing seasons

Parameter	Oct.	Nov.	Dec.	Jan.	Feb.	Mar.	Apr.	May.	Jun.	Mean/Total
Temperature (°C)										
2013-2014	16.5	8.2	1.9	1.7	1.6	11.5	16.4	23.0	24.7	11.7
2014-2015	16.2	8.1	0.4	2.0	3.7	10.8	14.9	21.7	26.1	11.5
30-year-average	16.1	8.8	1.8	-0.4	2.2	8.2	16.1	21.8	26.3	11.2
Precipitation (mm)										
2013-2014	20.5	33.9	1.2	0.0	16.2	0.1	20.7	43.2	98.6	234.4
2014-2015	7.2	21.9	0.6	6.1	10.5	2.5	83.7	59.1	73.1	264.7
30-year-average	36.5	16.2	8.2	5.7	8.5	15.3	27.4	46.6	78.8	243.2

1.2 Supplementary Table 2 The chlorophyll a fluorescence measured in four NILs, NG-JM204 and NG-JM206 (Bold), G-JM205 and G-JM208 (Italic) in the dark both under WI and DS conditions

Line	WI			DS		
	F_v/F_m *	*ABS/RC* *	*PI* *	*F'_v/F'_m* *	*ABS'/'RC* *	*PI'* *
**NG-JM204**	**0.823** a	**0.558** b	**5.362** d	**0.778** b	**0.749** c	**2.969** c
**NG-JM206**	**0.822** a	**0.557** b	**5.708** b	**0.776** b	**0.784** b	**3.338** b
Average	0.823	0.558	5.535	0.777	0.767	3.154
G-JM205	*0.824* a	*0.575* a	*5.641* c	*0.782* a	*0.816* a	*3.292* b
G-JM208	*0.817* b	*0.562* b	*5.823* a	*0.785* a	*0.803* a	*3.640* a
Average	0.821	0.569	5.732	0.783	0.81	3.466

* Letters after the data indicate significant difference at P = 0.05.
